# Cholinergic pathways in neural stem cell regulation and glioblastoma progression: Shared origins and mechanisms

**DOI:** 10.4103/NRR.NRR-D-25-00288

**Published:** 2025-07-05

**Authors:** Moawiah M. Naffaa

**Affiliations:** Department of Psychology and Neuroscience, Duke University, Durham, NC, USA

Neural stem cells (NSCs) and glioblastoma stem cells (GSCs) share a complex regulatory landscape in which cholinergic signaling plays a pivotal role in both neural development and tumor progression. While acetylcholine (ACh) regulates NSC quiescence and differentiation within neurogenic niches, glioblastoma cells exploit these pathways to enhance their adaptability and invasiveness. The involvement of muscarinic (M3) and nicotinic (α7) receptors in both cell types suggests that glioblastoma retains neural progenitor-like traits, contributing to its plasticity and resilience. This article explores the shared cholinergic mechanisms between NSCs and GSCs, highlighting their role in both neural development and glioblastoma progression.

**Dynamic role of cholinergic signaling in neural circuit regulation of neural stem cell fate and function:** Neural circuits are integral to the regulation of NSCs, modulating the microenvironment and signaling pathways that dictate NSC behavior (Song et al., 2016; Naffaa, 2024). Composed of interconnected neurons and glial cells, these circuits provide essential inputs that influence NSC quiescence, activation, and differentiation. By integrating diverse neurotransmitter signals, such as glutamate, ACh, gamma-aminobutyric acid, and neuropeptides, these circuits collectively shape the functional state of NSCs within their niche. For instance, excitatory signals from local neural circuits can promote NSC proliferation and differentiation into neurons, while inhibitory signals may maintain NSCs in a quiescent state (Song et al., 2016; Naffaa, 2024).

Cholinergic signaling is particularly vital for regulating quiescent NSCs located in the subventricular zone (SVZ) of the lateral ventricles, where both postnatal and adult neurogenesis occur. Within this region, NSCs can remain quiescent, awaiting specific extrinsic signals that prompt their activation and subsequent division (Naffaa and Yin, 2024). The release of ACh from cholinergic neurons profoundly influences the proliferation of these quiescent NSCs.

The interaction between ACh and its receptors is essential for transitioning NSCs from quiescence to active division, facilitating either self-renewal or differentiation into neuroblasts. M3 muscarinic receptors (M3Rs) play a key role in this process by mediating effects of ACh on quiescent NSCs. Upon activation, M3Rs initiate intracellular signaling cascades, primarily leading to inositol trisphosphate production and subsequent calcium release from the endoplasmic reticulum, an essential step in NSC proliferation (Naffaa and Yin, 2024).

Despite the established roles of cholinergic signaling in NSC proliferation within the SVZ, several critical questions remain. What is the functional importance of this signaling pathway in pathological conditions? How does cholinergic signaling differ in the adult human SVZ, where neurogenesis is not active? Addressing these questions warrants further investigation to better understand the therapeutic potential of targeting cholinergic pathways.

Beyond the SVZ, cholinergic signaling is pivotal in regulating NSCs in the hippocampus. Cholinergic neurons, primarily projecting from the basal forebrain, release ACh, which acts through nicotinic and muscarinic receptors on NSCs and their progeny (Song et al., 2016). This signaling pathway not only promotes NSC proliferation and differentiation but also enhances the integration of newly generated neurons into existing hippocampal circuitry. Such integration is essential for maintaining cognitive functions, including memory formation and spatial navigation.

Disruptions in cholinergic signaling can impair neurogenesis and are implicated in various neurodegenerative disorders, including Alzheimer’s disease, where cholinergic deficits are particularly pronounced (Chen et al., 2022). Understanding the underlying mechanisms that contribute to cholinergic dysfunction in these conditions is essential for informing potential therapeutic strategies. Insights into cholinergic signaling mechanisms in the hippocampus may pave the way for enhancing neurogenesis and addressing cognitive decline in neurodegenerative conditions.

**Cholinergic signaling in glioblastoma stem cells — tumor progression, microenvironment adaptation, and neuro-tumor connectivity:** Cholinergic signaling plays a critical role in the progression of GSCs, which are pivotal to the aggressive nature of glioblastoma. GSCs integrate into neural circuits and receive direct input from cholinergic neurons, actively promoting tumor invasion and growth (Venkatesh et al., 2019). The formation of functional neuron-GSC connections facilitates the expansion and survival of these cells within the brain microenvironment.

Evidence indicates that both cholinergic and glutamatergic neurons contribute to GSC progression, highlighting the intricate interplay between neuronal activity and tumor dynamics (Venkatesh et al., 2019; Tetzlaff et al., 2025). GSCs exploit these neuronal signaling pathways to enhance their proliferation, migration, and resistance to therapies, indicating that they do not operate in isolation but rather as part of a complex neuro-tumor ecosystem.

The connectivity between neurons and GSCs is dynamic, varying according to the state of the tumor cells. Notably, synaptogenic gene expression correlates with increased invasiveness, underscoring the adaptive capabilities of GSCs as they leverage neuronal inputs to sustain tumor growth and evade immune responses (Restaino et al., 2023). External stimuli, such as neuronal hyperactivity, can influence tumor progression by modulating neurotransmitter release, thereby strengthening neuron-GSC connectivity. This raises fundamental questions regarding how GSCs actively shape neuronal activity to maintain their survival. Do GSCs alter neurotransmitter homeostasis to create a permissive microenvironment, and if so, what are the underlying molecular mechanisms?

Cholinergic signaling plays a critical role in tumor dynamics beyond glioblastoma, with M3Rs (Tetzlaff et al., 2025) and nicotinic acetylcholine receptors (nAChRs) emerging as key regulators of GSC proliferation and invasion. Notably, while the α7 nAChR subtype facilitates communication between cholinergic neurons and GSCs, enhancing their responsiveness to cholinergic signaling, emerging evidence suggests that the α9 nAChR subtype may also contribute to tumor progression by regulating cell proliferation, survival, and invasion (Pucci et al., 2021). These interactions collectively drive tumor growth and infiltration into surrounding brain tissue, fostering a more aggressive tumor phenotype.

Interestingly, M3R overexpression has been implicated in the progression of other cancers, including colorectal cancers, where it promotes epithelial-to-mesenchymal transition, cellular proliferation, and resistance to apoptosis (Cheng et al., 2017). The shared involvement of M3Rs and nAChRs in glioblastoma and other malignancies suggests a broader oncogenic role for these receptors across diverse cancer types.

One hypothesis is that the tumor-promoting effects of M3Rs and nAChRs are mediated by their ability to enhance cellular plasticity, enabling cancer cells, including GSCs, to adapt to their microenvironment. This perspective aligns with recent findings that underscore the importance of cholinergic signaling in modulating tumor behavior beyond the central nervous system.

To further understand these dynamics, it is crucial to explore how neuron-GSC interactions in glioblastoma compare to those in peripheral cancers, where cholinergic signaling is also implicated. Could the mechanisms driving neuron-GSC interactions in glioblastoma contribute to malignancies in non-neural tissues?

Addressing these questions requires interdisciplinary research that bridges neuro-oncology and systemic cancer biology. Investigating how cholinergic signaling integrates with other molecular pathways in both central and peripheral cancers is essential. Does nAChR activation confer similar adaptive advantages across different tissue environments, or are there distinct mechanisms driving tumor progression in various contexts? By elucidating the broader role of cholinergic signaling in tumor progression, we can better understand the complex interactions between neural signaling and glioblastoma stem cells.

**Shared origins and mechanisms of glioblastoma and neural stem cells — a cholinergic crossroad between regeneration and malignancy:** Cholinergic signaling serves as a pivotal mediator in both NSC regulation and glioblastoma progression, highlighting a striking convergence between neurogenesis and tumor biology. This dual role raises fundamental questions about the shared developmental origins of glioblastoma and NSCs, particularly given their similar mechanisms of growth, plasticity, and microenvironmental interactions. Understanding these parallels could provide new insights into both tumor adaptation and neurodevelopment, revealing potential therapeutic targets.

**Figure 1:**
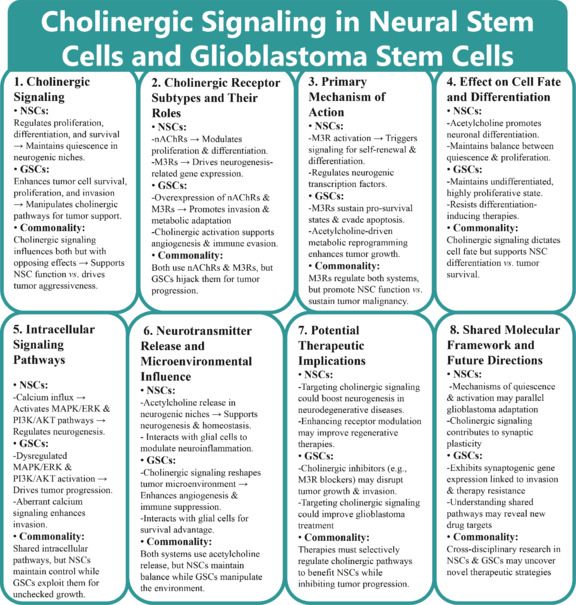
Cholinergic signaling in neural stem cells and glioblastoma stem cells. GSCs: Glioblastoma stem cells; M3Rs: muscarinic acetylcholine receptors; MAPK/ERK: mitogen-activated protein kinase/extracellular signal-regulated kinase; nAChRs: nicotinic acetylcholine receptors; NSCs: neural stem cells; PI3K/AKT: phosphatidylinositol 3-kinase/ protein kinase B.

From a developmental perspective, NSCs residing in neurogenic niches, such as the SVZ and hippocampus, rely on cholinergic signaling, particularly through M3Rs, to regulate their fate. Activation of these receptors triggers intracellular calcium signaling, promoting NSC proliferation and differentiation. nAChRs also play a crucial role in NSC biology, influencing neurogenesis through ligand-gated ion channels that mediate rapid synaptic transmission. These receptors contribute to neural development by regulating progenitor cell proliferation, migration, and differentiation. However, the same pathways that govern healthy neurogenesis appear to be hijacked by glioblastoma cells, which exploit cholinergic signaling to enhance their invasive and adaptive capabilities within the brain microenvironment. This functional overlap suggests that glioblastoma may, in part, retain the plasticity and responsiveness of NSCs, enabling its aggressive behavior (**Figure 1**).

**Figure 2:**
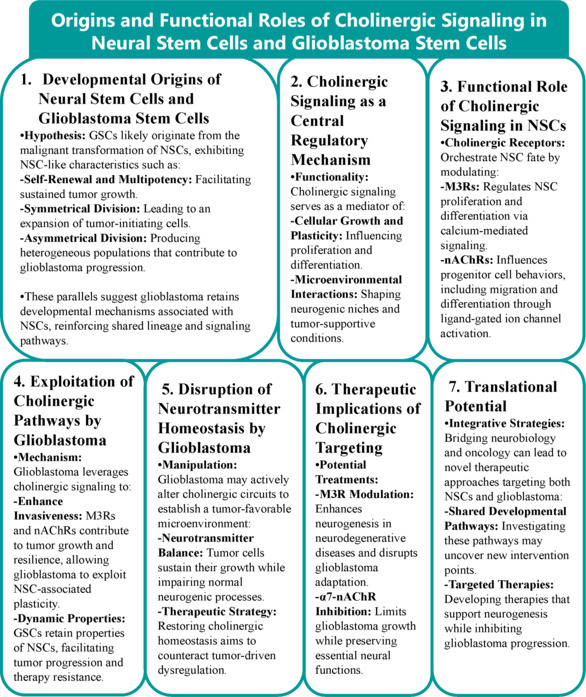
Developmental origins and cholinergic signaling in neural and glioblastoma stem cells: mechanisms of tumor progression and therapeutic implications. GSCs: Glioblastoma stem cells; M3Rs: muscarinic acetylcholine receptors; nAChRs: nicotinic acetylcholine receptors; NSCs: neural stem cells; α7-nAChR: alpha seven nicotinic acetylcholine receptor.

An intriguing perspective arises when considering whether glioblastoma and NSCs share not only mechanistic similarities but also a common cellular origin. Glioblastoma has long been proposed to arise from a population of neural progenitor-like cells, possibly NSCs that have undergone malignant transformation. GSCs exhibit key features of NSCs, including the capacity for self-renewal and multipotency. Importantly, both NSCs and GSCs undergo symmetrical and asymmetrical divisions, processes essential for maintaining homeostasis and tissue regeneration. Symmetrical division expands the stem cell pool, while asymmetrical division generates differentiated progeny. Dysregulation of these processes in glioblastoma likely contributes to its highly invasive and therapy-resistant nature. If so, then glioblastoma’s reliance on cholinergic pathways may reflect an intrinsic feature inherited from normal neurogenesis rather than an entirely novel adaptation. This hypothesis aligns with findings that glioblastoma cells exhibit synaptogenic gene expression linked to invasiveness, mirroring how excitatory neurotransmission influences NSC activity and fate decisions (**Figure 2**).

Given this potential commonality, targeting cholinergic signaling offers a unique therapeutic avenue. Modulating M3R activity could, on the one hand, enhance neurogenesis in neurodegenerative diseases while, on the other hand, serve to disrupt glioblastoma’s adaptive advantages. Nicotinic receptors, particularly the α7 subtype, are also implicated in glioblastoma progression, suggesting that selective antagonism could mitigate tumor growth while preserving essential neural functions (**Figure 2**). The challenge lies in selectively manipulating these pathways without impairing essential neural functions, necessitating further research into context-dependent cholinergic signaling.

Additionally, the impact of glioblastoma on neurotransmitter homeostasis remains an open question. Could glioblastoma actively reshape local cholinergic signaling to construct a tumor-favorable niche? If so, restoring or reprogramming neurotransmitter balance in the tumor microenvironment might serve as a novel strategy to counteract its progression. Understanding how glioblastoma co-opts neural circuits may thus hold the key to not only inhibiting tumor growth but also uncovering fundamental principles governing NSC function.

Ultimately, bridging neurobiology and oncology through the lens of cholinergic signaling provides a compelling framework for future research. Investigating these shared mechanisms could lead to innovative therapies that simultaneously target glioblastoma progression and promote neural regeneration. By integrating developmental neuroscience with cancer biology, we may uncover therapeutic strategies that transcend traditional disease boundaries, offering hope for both neurodegenerative conditions and malignant brain tumors alike.
